# Effect of Minimally Invasive Internal Arch Nailing Surgery on Tissue Traumatic Stress Response in Patients with Vertebral Fractures

**DOI:** 10.1155/2022/2375883

**Published:** 2022-08-16

**Authors:** Zuzhong Zhou, Zhixiang Chen, Liang Gao, Qizhao Sun, Wen Tang

**Affiliations:** Zibo Hospital of Shandong Yiyang Health Group, Zibo, Shandong 255051, China

## Abstract

**Objective:**

To analyze the influence of minimally invasive arch root nailing internal fixation surgery on tissue traumatic stress response in patients with vertebral fractures and explore the advantages of this treatment.

**Methods:**

One hundred and thirty-six patients with vertebral fractures admitted to our hospital from January 2020 to January 2022 were selected and divided into two groups based on the treatment method: the control group was treated with open arch root nail internal fixation surgery and the study group was treated with minimally invasive arch root nail internal fixation. The lumbar spine function, ODI, VAS, JOA score, complications, inflammation, and stress response were compared between the two groups.

**Results:**

After the operation, the ratio of intervertebral space and anterior edge height increased, and the Cobb angle decreased in both groups; the surgical incision, hospital stay, and operation time in the study group were shorter than those in the control group, and the intraoperative drainage volume and intraoperative blood loss were smaller than those in the control group (*P* < 0.05); before surgery, there was no significant difference in ODI and VAS scores between the two groups (*P* > 0.05). After surgery, the ODI and VAS scores in the two groups were significantly decreased, and the JOA score was significantly increased; complications occurred in the control group and the study. The incidence of complications in the study group was lower than that in the control group (*P* < 0.05); after surgery, compared with the control group, the serum TNF-*α*, CRP levels, and stress response indexes of the study group decreased more significantly (*P* < 0.05).

**Conclusion:**

Minimally invasive pedicle screw fixation has high safety and obvious advantages. The patient's stress response index and pain level are low, and it will not cause obvious damage to the patient. The postoperative lumbar spine function is significantly improved, which is beneficial to the patient's postoperative recovery. It is easy to operate, will not damage the thoracic and lumbar vertebrae significantly, and the fluoroscopy time is relatively short, and it has a good recovery effect. Therefore, minimally invasive internal arch nailing surgery can be used as the preferred treatment for patients with vertebral fractures.

## 1. Introduction

The spine is an important structure of the human body, supporting body weight, participating in human movement, and having a protective and shock-absorbing effect [1]. Vertebral fractures are bone destructive diseases induced by violence and trauma. Patients often suffer from neurological damage, which is very easy to be damaged under the action of high energy. Therefore, it will be combined with other organ damage, affecting the patient's normal movement and life seriously. It can even lead to paralysis, so early treatment is required, and the treatment is more difficult [2]. Currently, surgery is often used for treatment, which can effectively correct the damaged vertebral structures and can promote the improvement of spinal function [3]. Because of the reduced stability of lumbar spine fractures, damage to the spinal nerves can occur, and a treatment method with rapid postoperative recovery and few complications needs to be explored to ensure safe treatment [4]. Using traditional open pedicle screw fixation, the incision is long, and the time for freeing muscles, ligaments, and fascia is relatively long. Repeated stretching is required during the operation, so the patient will experience muscle ischemic necrosis and a degree of pain in the patient. Obviously, postoperative complications are prone to occur [5]. In recent years, various minimally invasive techniques have developed rapidly. Currently, minimally invasive pedicle screw internal fixation is used to restore the height of the compressed vertebral body in patients, which can make up for the insufficiency of traditional treatment and surgery [6]. In this study, we chose open and minimally invasive internal fixation with an arch nail to analyze the effect on the stress response of patients with vertebral fractures, hoping to improve the function of the lumbar spine and improve their ability to live, respectively, as reported below.

## 2. Materials and Methods

### 2.1. General Materials

One hundred and thirty-six patients with vertebral fractures admitted to our hospital from January 2020 to January 2022 were selected and divided into two groups, the control group and the study group, based on the treatment method. All subjects in this study signed informed consent, and there were no cases lost to follow-up. Inclusion criteria were (1) patients meeting the diagnostic criteria of vertebral fracture [7]; (2) patients with spinal canal occupancy less than 1/3 of the sagittal diameter of the spinal canal; (3) patients with single-segment fractured vertebrae; (4) patients without spinal canal decompression and neurological symptoms. Exclusion criteria were (1) patients with old fractures; (2) patients with unconsciousness; (3) patients with nerve injury; (4) patients with contraindications to internal fixation surgery; (5) patients with severe cardiovascular and cerebrovascular diseases. In the control group (*n* = 68), 35 males and 33 females, age 20–69 years, mean age (45.69 ± 2.37) years, mean duration of illness (4.55 ± 0.77) days, causative factors: 18, 30, and 20 cases of heavy object injury, traffic accident, and fall from height, respectively; in the study group (*n* = 68), 36 males and 32 females, age 20–68 years, mean age (45.67 ± 2.35) years old, mean duration of illness (4.57 ± 0.76) days, causative factors: 19, 28, and 21 cases of heavy object injury, traffic accident, and fall from height, respectively.

### 2.2. Methods

#### 2.2.1. Open Endoprosthesis Treatment

The patient was anesthetized with tracheal intubation, and the incisional approach was chosen for the posterior median position of the spine. According to the preoperative imaging features, the center was designated as the position of the spinous process of the diseased vertebral body, and the skin tissue was incised layer by layer to fully reveal the normal and injured vertebral bodies. Two pedicle screws were placed into the adjacent vertebral body under C-arm fluoroscopy to fully stretch the injured vertebral body and restore the height of the anterior edge of the vertebral body. For the connecting rod installation, tighten the nail cap, lock the connecting rod after completing the repositioning, aseptically treat the wound, leave a drainage tube in place, and then suture the wound.

#### 2.2.2. Minimally Invasive Internal Fixation Treatment with the Pedicle Nail

Two cushions are placed on the buttocks and chest, respectively, and the abdomen is suspended so as not to compress it. The injured vertebral body was positioned under X-ray, and the position of the kerf pins for the vertebral arch was marked on the body surface, and then 1.5 cm incisions were made with each of the four arch attachments adjacent to the two vertebral bodies. The screws were placed on the lateral side of the pedicle and slowly punctured to the medial axis of the pedicle, and the tip of the needle at the inner edge of the pedicle and the tip of the needle at the posterior edge of the vertebral body were shown on the orthogonal and lateral views, respectively, followed by a 1 cm puncture, and then a guide wire was placed and the pedicle screws were screwed into the vertebral body under the guidance of the guide wire, followed by sequential placement of the remaining screws, observation of the screw fixation and positioning status by X-ray, placement of a fixation rod in the caudal groove of the pedicle screws, fixation of the screw cap The wound was sutured and disinfected. Postoperative monitoring of patients, antibiotic treatment for infection, fluid replacement to maintain water and electrolyte balance, guidance for patients to turn over, and effective management of lungs and urethra, to avoid pulmonary infection and systemic infection, and observation of the condition of the drainage tube. The patients were followed up after surgery until their spinal function returned to normal.

### 2.3. Observation Indexes

#### 2.3.1. Vertebral Space, Anterior Margin Height Ratio, Cobb Angle

The injured vertebral segment was centered, X-ray and CT frontal and lateral radiographs were taken for diagnosis, vertebral space and Cobb angle were recorded, and the anterior margin height ratio was calculated.

#### 2.3.2. Surgical Results

The surgical incision, intraoperative drainage and blood loss, hospitalization, and operative time were recorded.

#### 2.3.3. VAS [8]

The visual analog scale (VAS) was applied to evaluate the pain level of patients with a total score of 10, with 7–10 indicating severe pain, 4–6 indicating moderate pain, 1–3 indicating mild pain, and 0 indicating no pain.

#### 2.3.4. JOA [9]

The Japanese Orthopedic Association (J0A) criteria were applied to assess patients' lumbar spine function, which contains four components: bladder function (0–6 points), degree of life limitation (0–14 points), subjective symptoms (0–9 points), and clinical signs (0–6 points), and the scores were positively correlated with lumbar spine function.

#### 2.3.5. ODI Score [10]

The dysfunction index questionnaire (ODI) was applied to evaluate the patient's life functions, including walking, sitting, traveling, sleeping, pain, lifting, living, self-care, and standing functions, and the scores were positively correlated with the degree of dysfunction.

#### 2.3.6. Complications

The number of cases of incisional infection, muscle injury, incisional bleeding, internal fixation displacement, and bone discontinuity was counted, and the incidence was calculated.

#### 2.3.7. Inflammatory Factors and Stress Reactions

3 ml of fasting venous blood was drawn, centrifuged, and processed to see at 3500 rpm, and serum C-reactive protein (CRP), tumor necrosis factor-*α* (TNF-*α*), norepinephrine (NE), and cortisol (Cor) levels were detected by applying enzyme-linked immunoassay [11], and serum creatine kinase (CK) was determined by applying electrochemiluminescence method [12]. Levels and the related operations were performed strictly according to the instructions.

### 2.4. Statistical Methods

Statistical SPSS22.0 software was used for data analysis. If the data conformed to a normal distribution, the complication count data were described by the composition ratio and rate, and the chi-square test was used for the analysis of differences between groups. Intervertebral space, anterior edge height ratio, Cobb angle, surgical results, VAS, JOA, ODI score, inflammatory factor levels, stress response, and other measurement data are expressed as (mean ± standard deviation), taking *P* < 0.05 to be comparable between the two groups, the researchers used the graphing software GraphPad Prism8.

## 3. Results

### 3.1. Comparison of General Information between the Two Groups

The data of gender, age, injured vertebral site, and time of injury were comparable between the two groups (*P* > 0.05), the details can be seen from Table 1.

### 3.2. Comparison of Vertebral Space, Anterior Margin Height Ratio, and Cobb Angle between the Two Groups

Preoperatively, there was no statistically significant difference in the vertebral space, anterior margin height ratio, and Cobb angle between the two groups (*P* > 0.05), but postoperatively, the vertebral space and anterior margin height ratio increased and Cobb angle decreased in both groups. The results are as shown in Table 2.

### 3.3. Comparison of Surgical Outcomes between the Two Groups

The surgical incision, hospital stay, and operation time in the study group were shorter than those in the control group, and the intraoperative drainage and intraoperative blood loss were smaller than those in the control group (*P* < 0.05). The results are shown in Table 3.

### 3.4. Comparison of VAS, JOA, and ODI Scores between the Two Groups

**Figure 1:**
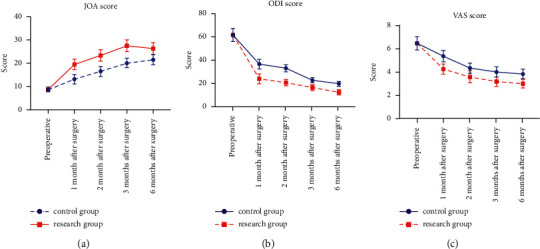
Comparison of JOA and ODI scores between the two groups.

Before surgery, there was no significant difference in VAS, JOA, and ODI scores between the two groups (*P* > 0.05). After surgery, ODI and VAS scores were significantly lower and JOA scores were significantly higher in both groups, but the magnitude of change was greater in the study group, and the difference was statistically significant (*P* < 0.05) when compared between the groups. The detailed analysis is shown in Figure 1.

### 3.5. Comparison of Complication Rates between the Two Groups

**Figure 2:**
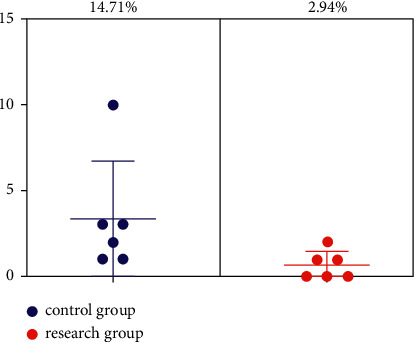
Comparison of complication rates between the two groups.

The complication rates in the control and study groups were 2.94% and 14.71%, respectively, and the complication rates in the study group were lower than those in the control group (*P* < 0.05), as shown in Figure 2.

### 3.6. Comparison of Inflammatory Factor Levels between the Two Groups

**Figure 3:**
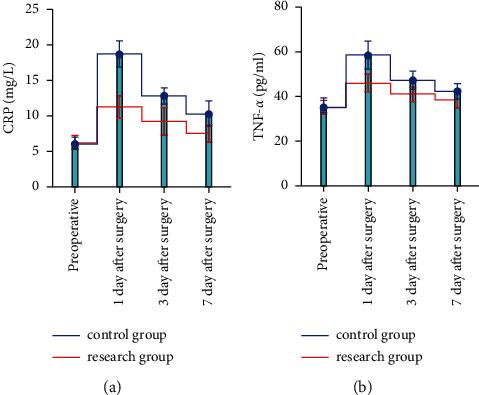
Comparison of inflammatory factor levels between the two groups.

Before surgery, there was no significant difference in the levels of inflammatory factors between the two groups (*P* > 0.05). After surgery, the levels of inflammatory factors were significantly reduced in both groups, but the reduction in serum TNF-*α* and CRP levels was greater in the study group compared with the control group (*P* < 0.05), as shown in Figure 3.

### 3.7. Comparison of Stress Reaction Indexes between the Two Groups

**Figure 4:**
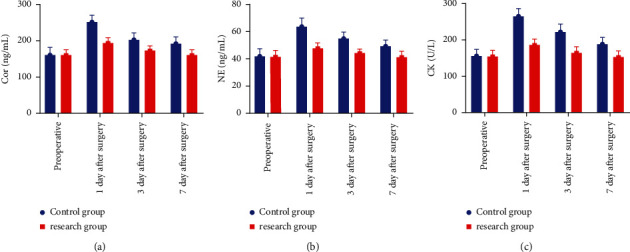
Comparison of stress response indicators between two groups.

Before surgery, there was no significant difference in stress response indexes between the two groups (*P* > 0.05). After the operation, the stress reaction indexes were significantly reduced in both groups, but the reduction in stress reaction indexes was greater in the study group compared with the control group (*P* < 0.05), as shown in Figure 4.

## 4. Discussion

Patients' vertebral fractures have limited mobility and severe pain, which affects their life functions and is treated mainly by surgical means [13]. The application of traditional surgical treatment can effectively correct and reset the fracture end, but it has more complications and can cause significant damage to the patient with high bleeding, which affects the patient's postoperative recovery [14]. Open pedicle nail internal fixation treatment can strip the paravertebral muscles in a large area, which can cause serious damage to the patient's spinal nerve function, and the patient will be accompanied by low back stiffness and pain symptoms, and the prognosis is not ideal [15]. In addition, some patients have a more pronounced degree of pain and restricted function of the organism, reducing the patient's muscle function and showing obvious symptoms of muscle scarring and fibroadenoma [16].

At present, medical technology continues to progress and develop, and minimally invasive techniques are becoming more and more advanced and widely used in clinical treatment, with shorter surgical and postoperative recovery times, less postoperative storage in patients, and no significant damage to patients, so minimally invasive surgical treatment has more obvious advantages compared with traditional surgical treatment [17]. Positioning with the aid of a C-arm X-ray machine can clarify the length and location of the surgical incision and does not require, which can reduce intraoperative bleeding and can avoid damage to the muscles and medically induced injuries, with a short postoperative recovery time. Minimally invasive internal fixation of the pedicle nail has a greater improvement in patient prognosis compared to traditional treatment, but this treatment also has limitations due to the limited length of the incision [18]. Therefore, the operator needs to be technically competent and needs to adjust the angle of the approaching needle under X-ray guidance to avoid displacement and deviation of the guide pin, and if abnormalities occur the positioning pin should be reinserted in the bone tract to use it as a guide for vertebral body posterior margin expansion [19].

After surgical trauma patients will have a significant stress response, and the degree of the stress response can be reflected clinically by the serum CRP index [20]. In the case of tissue trauma and inflammatory reaction, serum TNF-*α* expression is enhanced, inflammatory factor activity is increased, and a series of inflammatory cascade reactions will be accompanied, and the body will experience elevated levels of stress hormones in response to external stimuli [21]. Serum Cor and NE indicators can also reflect the status of the body's stress response with a high sensitivity. Surgery can cause systemic stress response and muscle damage, which is a special stressor, and the CK levels of skeletal muscle, cardiac muscle, brain, and other tissue cells are significantly elevated, which can be used as an important indicator to determine muscle damage [22]. Patients experience significant muscle strain and fracture trauma during open arch root nail internal fixation surgery treatment, and plasma CK levels are significantly elevated [23]. Open arch root nail internal fixation surgical treatment requires paravertebral muscle distraction under the guidance of an automatic retractor, and paravertebral muscle ischemia is evident, which will further reduce the risk of lumbar back muscle ischemia and increase the pain level of patients [24, 25]. The application of minimally invasive pedicle screw internal fixation can reduce the risk of denervation atrophy, the VAS score is significantly reduced, the intraoperative drainage volume and blood loss are reduced, and the patient's lumbar spine function and daily living ability are significantly restored. The results of this study are consistent with others. The results of scholars' research are consistent, and it is a treatment method worthy of promotion [26]. This study analyzed the effect of minimally invasive pedicle screw internal fixation on the tissue trauma stress response of patients with vertebral fractures. However, due to the relatively small number of included samples and the short study time, it will interfere with the treatment effect to a certain extent. Therefore, it is necessary to expand the study sample, prolong the study period, and improve the feasibility and accuracy of the study.

## 5. Conclusion

Minimally invasive pedicle screw fixation has high safety and obvious advantages. The patient's stress response index and pain level are low, and it will not cause obvious damage to the patient. The postoperative lumbar spine function is significantly improved, which is beneficial to the patient's postoperative recovery. It is easy to operate, will not damage the thoracic and lumbar vertebrae significantly, and the fluoroscopy time is relatively short, and it has a good recovery effect. Therefore, minimally invasive pedicle screw fixation can be the first choice for the treatment of patients with vertebral fractures.

## Figures and Tables

**Table 1:** Comparison of general information between the two groups.

Group	n	Gender		Average age	Injured vertebral body parts				Time of injury
		Female	Male		*T*_11_	*T*_12_	*L*_1_	*L*_2_	
Control group	68	35	33	50.32 ± 3.24	7	22	26	13	5.32 ± 1.25
Study group	68	36	32	50.33 ± 3.22	13	13	27	15	5.36 ± 1.24
*t*	—	0.030		0.018	0.269				0.187
*P*	—	0.864		0.986	0.864				0.852

**Table 2:** Comparison of vertebral space, anterior margin height ratio, and Cobb's angle between the two groups $\left[\bar{x}\pm s\right]$.

Group	n	Vertebral clearance (mm)			Leading edge height ratio (%)			Cobb angle (°)		
		Preoperative	3 months after surgery	6 months after surgery	Preoperative	3 months after surgery	6 months after surgery	Preoperative	3 months after surgery	6 months after surgery
Control group	68	5.49 ± 0.60	8.93 ± 0.70	9.75 ± 0.89	53.55 ± 9.67	85.69 ± 11.38	88.39 ± 12.35	18.26 ± 3.32	7.32 ± 1.37	7.49 ± 1.78
Study group	68	5.51 ± 0.64	9.10 ± 0.75	9.86 ± 0.99	52.37 ± 10.27	89.64 ± 11.37	90.37 ± 10.62	18.35 ± 3.23	7.75 ± 1.58	7.24 ± 1.43
*t*	—	0.188	1.366	0.681	0.690	2.025	1.002	0.160	1.696	0.903
*P*	—	0.851	0.174	0.497	0.492	0.045	0.318	0.873	0.092	0.368

**Table 3:** Comparison of surgical outcomes between the two groups $\left[\bar{x}\pm s\right]$.

Group	Number of cases	Surgical incision (cm)	Length of hospitalization (d)	Surgery time (d)	Intraoperative drainage flow (ml)	Intraoperative blood loss (ml)
Control group	68	15.25 ± 1.55	11.88 ± 2.35	126.52 ± 15.24	17.32 ± 1.26	126.31 ± 3.25
Intervention group	68	2.37 ± 0.26	8.23 ± 1.03	93.47 ± 3.24	6.53 ± 1.21	85.46 ± 2.05
*t*	—	67.579	11.731	17.492	50.934	87.666
*P*	—	<0.001	<0.001	<0.001	<0.001	<0.001

## Data Availability

The data are available on request from the corresponding author.
